# Global and Local Attention-Based Free-Form Image Inpainting

**DOI:** 10.3390/s20113204

**Published:** 2020-06-04

**Authors:** S. M. Nadim Uddin, Yong Ju Jung

**Affiliations:** College of Information Technology Convergence, Gachon University, Seongnam 1342, Korea; nadim@gc.gachon.ac.kr

**Keywords:** free-form mask, image inpainting, mask update, convolutional neural networks (CNN), attention module

## Abstract

Deep-learning-based image inpainting methods have shown significant promise in both rectangular and irregular holes. However, the inpainting of irregular holes presents numerous challenges owing to uncertainties in their shapes and locations. When depending solely on convolutional neural network (CNN) or adversarial supervision, plausible inpainting results cannot be guaranteed because irregular holes need attention-based guidance for retrieving information for content generation. In this paper, we propose two new attention mechanisms, namely a mask pruning-based global attention module and a global and local attention module to obtain global dependency information and the local similarity information among the features for refined results. The proposed method is evaluated using state-of-the-art methods, and the experimental results show that our method outperforms the existing methods in both quantitative and qualitative measures.

## 1. Introduction

Image inpainting or hole filling is a task for generating plausible alternative contents for the missing regions of a corrupted image, and this particular problem has been considered to be one of the most challenging tasks in computational photography. An image can be corrupted with holes, texts, or unwanted objects that can be removed and/or filled in with novel contents using available information from the image itself or from different images through image inpainting. However, the main challenge of image inpainting lies in generating plausible missing content with realistic textures and consistent structures.

Prior to deep-learning-based approaches, most of the image inpainting methods were focused on non-learning techniques (i.e., information propagation from hole boundaries, copying similar patches from the background) [1,2,3,4,5,6,7,8]. However, it is impossible to generate novel contents or fill in larger holes with these methods because the generated contents are often inconsistent with the remaining regions of the image. As a result, non-learning-based techniques are ineffective in addressing the inpainting problem in a larger context. With the emergence of convolutional neural network (CNN)-based approaches, it has become possible to generate novel contents for missing regions, even for comparatively larger holes [9,10,11,12,13,14]. Despite this, CNN-based image inpainting techniques tend to generate blurry content, boundary artifacts, and unrealistic content. Adversarial supervision [15], i.e., generative adversarial networks (GANs) over CNN models, have provided additional guidance on content generation, and hence, this type of coupled model, i.e., a CNN for generation and GAN for supervision, can generate more refined and visually aesthetic results. However, generating consistent structures and realistic textures are still regarded as open challenges to be tackled.

**Figure 1:**
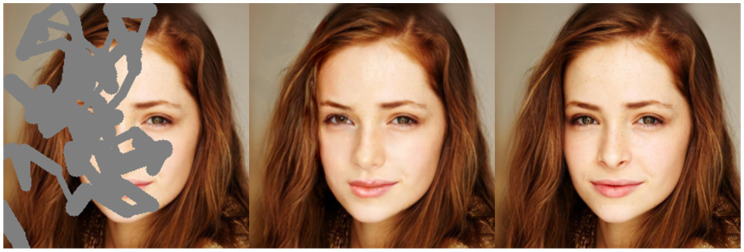
Example of free-form image inpainting. From left, corrupted image with a free-form mask, inpainted image obtained by our proposed method, and respective ground truth. Please note that due to the availability of global and local attention, our model generates visually plausible inpainting results.

Early approaches [9,10,11,12,13,14] use only local contextual information, i.e., local similarities among the feature patches, for the inpainting tasks. However, local contextual information from a CNN can provide local similarity information that can be used to refine the inpainted region and cannot identify global dependencies essential for structural consistency. In the case of free-form image inpainting, holes can appear in any shape and at any location in the image (see Figure 1). Hence, it is important to have both global and local contextual information to ensure visually consistent inpainting results. However, the benefits of incorporating global information have not been discussed in most of the recent CNN-based methods focusing on free-form image inpainting [16,17,18,19,20]. These approaches use only local information provided by convolution operations and cannot avoid the obvious texture and structure discrepancies caused by a lack of global information of the feature maps. Moreover, recent free-from image inpainting approaches [14,16,17,18,19,20] use attention mechanisms [14,16] to provide feature similarity information for feature reconstructions, or mask update mechanisms [16,17] for generating missing pixel values based on either layer gating or update rules. In all existing methods for free-form image inpainting, the feature maps extracted from the corrupted image contain hole features as well and can lead to inconsistent inpainting results. Based on results of recent approaches [16,17], it is evident that mask features contribute heavily when calculating similarities at the patch level, and need to be pruned when applying an attention mechanism. However, due to the mask features, the attention mechanisms or mask update mechanisms cannot efficiently ‘pick-up’ the most contributing image features and fail to generate convincing inpainting results. As a result, free-form image inpainting tasks face two crucial problems, namely how to incorporate global contextual information in the inpainting model and how to handle the mask features.

In this paper, we address these particular problems to facilitate free-form image inpainting tasks. If global contextual attention is integrated with inpainting models along with local similarity information while incorporating an efficient mask feature pruning mechanism, the inpainting results should have more stable structural consistency and more realistic textures. Contextual information from an attention mechanism at the global level can provide structural consistency for the missing region, and local similarity information from a local attention mechanism can provide a smooth texture transition of the inpainted content with the background image. Moreover, for free-form image inpainting, if the mask features are pruned from both the global and local features (i.e., the feature maps and image patches), both global and local attention mechanisms can efficiently calculate the features that contribute the most to reconstructing the missing regions and can effectively select the best candidate features for an image inpainting task. Hence, we propose two novel attention modules, namely a mask pruning-based global attention module that calculates the dependencies among features at the global level (i.e., feature maps) with a mask pruning mechanism, and a global and local attention module that calculates both the global dependencies and local similarities at the local level (i.e., image patches). For brevity, the proposed mask pruning-based global attention module and global and local attention module are referred as MPGA module and GLA module respectively throughout the paper.

Specifically, we adopt a coarse-to-refinement approach similar to those in [14,16,20] to divide the inpainting process into two stages: (1) a coarse network for a coarse or rough estimation of the missing regions and (2) a refinement network for a refinement of the generated coarse contents. Because the coarse network generates a rough estimation of the overall structure, it is logical to integrate global information in this stage to achieve a robust and more stable structural consistency. We integrated the proposed MPGA module in the attention branch of the coarse network to provide global contextual information. Moreover, the proposed MPGA incorporates mask update mechanisms based on pruning out less important features at the global level (i.e., feature maps). As a result, the final feature map from the proposed MPGA module contains the most contributing features for a reconstruction of the coarse estimation. The refinement network takes the coarse output as an input and generates a detailed and refined output of the missing regions based on a calculation of both the global and local attention. Because a refinement network deals with generating a refined output, we integrate our proposed GLA module in the attention branch of the refinement network.

The proposed model has been evaluated with several existing state-of-the-art methods for irregular-sized holes. Specifically, it was trained with three popular datasets used for image inpainting tasks, namely Places365 [21], ImageNet [22], and CelebA-HQ [23]. During the experiments, the qualitative and quantitative results reveal that our model outperforms the existing state-of-the-art models.

This paper is organized into five sections. Section 2 discusses related studies on both traditional and learning-based image inpainting methods. Section 3 describes the proposed inpainting model along with the detailed descriptions of the two proposed attention modules, i.e., the MPGA module and GLA module. Section 4 outlines the experimental setups applied and provides a comparison with existing methods and a description of the feasibility of the proposed modules. Section 5 discusses possible future directions of the proposed approach and provides some concluding remarks.

## 2. Related Studies

Image inpainting methods can be generally divided into two major variations: traditional methods and learning-based methods. Traditional methods generally depend on patch- or diffusion-based approaches. Learning-based methods generally depend on learning semantics of the image using deep learning-based models and are often guided by the GANs. Attention-based methods can be regarded as a recent research focus on learning-based inpainting methods. Attention-based methods provide additional semantic information for a better image inpainting process.

### 2.1. Traditional Image Inpainting

Traditional non-learning-based methods [1,2,3,4,5,6,7,8,24,25,26,27,28,29,30] fill in missing regions by propagating neighboring information or copying information from similar patches of the background. Diffusion-based methods generally depend on variational methods and copy the surrounding background pixels directly into the hole regions. However, these methods can deal with only small holes (i.e., scratches or texts) and cannot generate novel content or handle large holes.

Patch-based methods focus on finding the most similar patches from the background and paste similar patches in the hole region. These methods can fill up larger holes and work well with stationary textures or plain structures. In [26], a bidirectional patch similarity method was proposed to model non-stationary visual data for image inpainting tasks. However, the patch similarity computation was expensive in [26], and hence in [27], a fast nearest-neighbor field method was proposed, with significant improvement shown in terms of speed and practical values for image editing application [14]. However, these methods do not work well with non-stationary textures such as natural scenes and complex images requiring generation of novel contents.

Moreover, diffusion- or patch-based methods can work with stationary textures or repetitive structures but do not perform well with larger holes or complex scenes. With the emergence of a CNN [31], a major shift of interest has been directed toward learning-based image inpainting methods.

### 2.2. Learning-Based Image Inpainting

Recent deep learning-based approaches [9,10,11,12,13,14,18,32,33,34,35,36,37,38] mostly focus on rectangular holes for image inpainting tasks. These methods generate plausible inpainting results by learning the semantics of the data based on extensive reference images for the learning process. Pathak et al. [9] first proposed a deep-learning-based inpainting model for generating novel contents for a 64×64 center hole region in a 128×128 image and a GAN for supervision. However, this model [9] performs poorly in generating fine details in an inpainted region. Iizuka et al. [10] proposed global and local discriminators for better supervision where the global discriminator supervises the entire content generation process, and the local discriminator supervises local consistency. However, it [10] requires post-processing to ensure consistency near the hole boundaries. Yan et al. [12] proposed a shift operation for gradually shifting the encoder features of the known region as an estimation of the missing parts using a guidance loss. However, a shift operation [12] requires a computationally expensive optimization process and trade-offs between the quality and speed.

Most of the methods in a learning-based approach depend on either adversarial losses or explicit optimization processes for content generation. Moreover, image inpainting in real applications contain free-form or irregular holes that require different optimization processes or ’attention mechanisms’ compared with rectangular holes. As a result, research interests have shifted more toward free-form image inpainting.

### 2.3. Attention-Based Image Inpainting

Recent approaches [16,17,19,20,39] have focused more on irregular shaped holes because free-form holes are more frequent and practical for use in image inpainting tasks. Most of the newer methods incorporate either mask update processes [16,17,39] or additional inputs [19,20]. In [17], a new mask update mechanism was proposed based on a new convolution scheme called a partial convolution, where after each convolution, the mask update procedure denotes the mask location as either valid or invalid based on whether the convolution is able to condition its output on at least one valid input. However, the mask update method is heuristic-based and validates the mask location based on predefined rule. In [16], a soft gating mechanism-based mask update method was proposed for free-form image inpainting tasks. By incorporating the layer gating mechanism, [16] implemented a soft mask update instead of the hard mask update proposed in [17]. In [19], edges were used as additional guidance for hallucinating structures before the content generation process. The edge generator predicts an edge map and uses it as an additional input for the content generation. However, because this method [19] first tries to hallucinate the edges for a structure reconstruction, the final inpainting result often contains blurry textures and artifacts due to the incorrect estimations of the edges. In [39], a conditional variational autoencoder (CVAE)-based approach was proposed while introducing a short + long attention module for capturing the spatial context as well as the feature–feature context. The short + long attention module uses a self-attention map within the decoder layer as well as an attention map between the encoder and decoder layers. The model uses a dual pipeline architecture with a short + long term attention module to capture both spatial contexts through intra-layer self-attention and refined features using an inter-layer attention mechanism. However, the original self-attention module cannot capture the local information owing to the presence of mask values and hence, inpainted results in [39] often contain inconsistent structures and texture mismatch. In [20], a method is proposed that uses the relative total variation-based smoothed structure images as additional structural guidance. With this method, a global structure image is first predicted as a coarse output and the structure image is then used to fine-tune the final results. However, the model uses an additional input comparable to that in [19]. In [40], a fusion technique is proposed for generating multiple alpha composition maps along with adjustable loss constraints. However, this method [40] depends on explicit blending mechanism (i.e., background image with the inpainting results using a fusion method), which is prone to poor texture transition and structural inconsistency.

Most of the existing methods do not consider the explicit relationships among features, specifically in both global and local feature aspects, and thus perform comparatively poorly in challenging scenes. Moreover, most of these methods disregard the effect of the hole features in the attention mechanisms, which can lead to inconsistent content generation. In this study, we leverage the global and local correlation among features as well as patches for content generation and refinement of the details by proposing two new attention modules, namely a MPGA module and a GLA module.

## 3. Proposed Model

**Figure 2:**
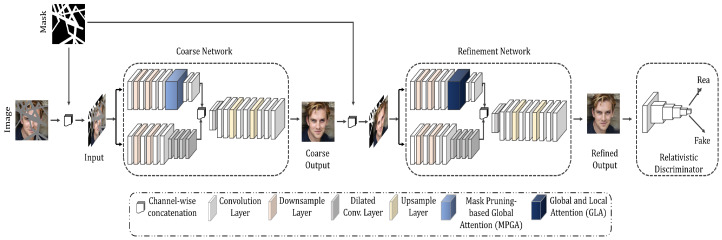
Overall architecture of the proposed model. The proposed model has two stages, namely a coarse network and a refinement network. Both the coarse and refinement networks have two branches, namely a regular branch and an attention branch. The coarse network calculates the global dependencies among the features and prunes out mask features using the proposed MPGA module, generating a rough or coarse inpainted image. The refinement network takes the coarse output as the input and calculates both global and local similarities using the proposed GLA module and produces a refined inpainting result.

Our proposed model consists of two stages, namely a coarse network and a refinement network, as shown in Figure 2. We construct our baseline inpainting network by adopting the coarse and refinement network architectures from [14] with several major modifications. For generating a coarse output, the previous study [14] uses a simple encoder-decoder network that outputs a blurry estimation of the inpainted regions. However, the coarse network in [14] does not have any additional guidance or attention mechanism to capture important feature dependency information. Instead of using a simple encoder-decoder network for the coarse output, we design a coarse network with an additional attention branch, along with the regular branch, in the encoder that can capture the structural consistency by calculating global correlation among features at the global level. Owing to the proposed MPGA module in the attention branch of the coarse network, our coarse network produces blurry yet structurally consistent estimation. The MPGA module is built upon the self-attention mechanism proposed in [41]. The self-attention mechanism [41] works plausibly for capturing global-level dependencies among features as it calculates global correlation information among features by ‘attending’ important features in a global view. However, in the cases of image inpainting, features contain hole regions. A simple global correlation-based attention mechanism will fail to guide the inpainting model to ‘attend’ important features while applying the attention mechanism. Hence, we propose a simple yet effective mask pruning mechanism in the MPGA module that can prune out ‘less important’ features before applying the attention mechanism. Moreover, the previous study [14] uses the ‘contextual attention’ module that calculates the patch-level similarity based on the inner product similarity for local-level refinement. However, only local-level refinement can generate ambiguous and repetitive contents as there is no global-level refinement. Instead of using only local-level similarity-based attention mechanism, we propose a GLA module that is integrated into the attention branch of the refinement network. The GLA module calculates both global-level and local-level feature dependencies, which provide local-level refinement and global-level structural consistency.

### 3.1. Coarse Network

The coarse network is based on an encoder-decoder network with a MPGA module. The MPGA module calculates the global dependencies among the features and prunes out the less important features for a robust estimation of both the structures and textures. Section 3.1.1 provides an overview of the coarse network architecture, and Section 3.1.2 explains the proposed MPGA module.

#### 3.1.1. Coarse Network Architecture

The coarse network consists of two separate branches, namely a regular branch and an attention branch in the encoder segment. The coarse network takes a normalized 4×256×256 image as an input (three color channels and one mask channel). Specifically, as shown in Figure 2, an input image with holes $I_{in}$ and a binary mask *M* are concatenated channel-wise and then fed into both the regular and attention branches of the coarse network. Please note that the binary mask contains either one or zero as pixel values, where a zero value indicates a hole pixel.

The regular branch of the coarse network uses 3×3 convolution layers with dilated convolution layers with a kernel size of 3×3 and rates of 2, 4, 8, and 16 to achieve large receptive fields that contribute to a better feature extraction. The parallel attention branch of the encoder segment contains the MPGA module. The output from both the regular branch and the attention branch are concatenated channel-wise and fed into a single decoder. The decoder outputs a coarse inpainted image $I_{coarse}$ with the same size as input (see Appendix B.1 for the coarse network architecture).

We chose to generate irregular masks on the fly during training to introduce more diversity and robustness to avoid an over-fitting. For a coarse reconstruction, we use a weighted sum of a $L_{1}$ loss and structural similarity (SSIM) loss explicitly.

#### 3.1.2. Mask Pruning-Based Global Attention

**Figure 3:**
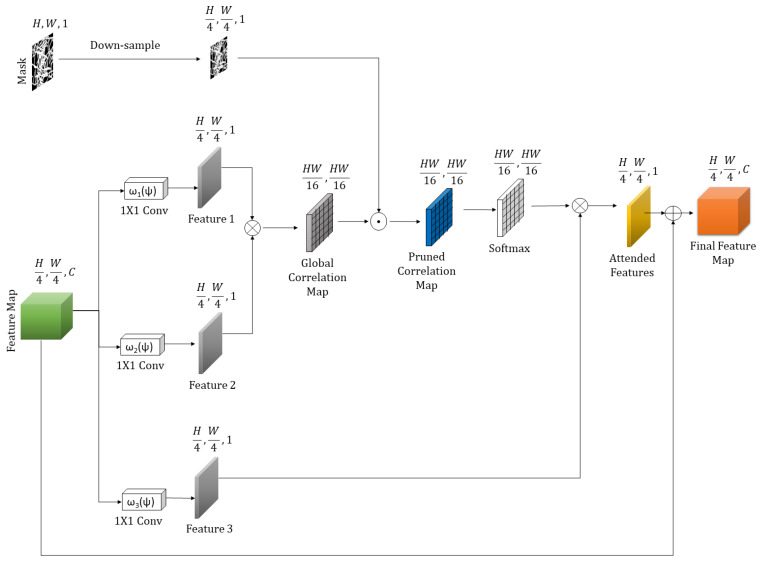
Overview of mask pruning-based global attention module. The module takes feature maps and a binary mask as input. The module calculates the global correlation among features and prunes the mask values from the correlation map. It then chooses the most contributing features from the pruned features and reconstructs the final feature map.

The mask pruning-based global attention (MPGA) module aims to achieve a better global semantic relationship among the encoded features. The proposed MPGA module is based on the self-attention mechanism proposed in [41]. The self-attention mechanism [41] uses information from the feature maps to generate global attention scores by performing a softmax calculation on the per-pixel correlation values. Then, the attention scores are multiplied with the feature map to highlight the most important regions in the feature map for further calculation. However, in the case of inpainting, integrating the original self-attention mechanism for global attention is insufficient due to the presence of mask values that contain no pixel information. To achieve a better global attention mechanism, it is important to have a robust pruning of the mask values in the feature map while calculating the global attention score. Figure 3 shows an abstract view of the MPGA module.

To calculate the global correlation among the feature values, we use the same feature map following a previous study [41] and apply the mask features for the pruning. Specifically, the input feature map of the proposed module comes from the previous hidden layer (i.e., the output feature map from the 6th convolution layer of the attention branch in coarse network, as shown in Figure 2). We extract three feature maps from the input feature map as per [41]. We use the two output feature maps (i.e., Feature 1 and Feature 2 in Figure 3) to calculate the global correlation among the features.

Formally, the proposed MPGA module takes the input feature map and mask as inputs. We denote the input feature map and mask values as ψ and *M*, respectively. The mask is resized to match the spatial dimension of the input feature map using the nearest neighbor interpolation method.

We first calculate the global correlation map ρ between $\omega_{1}\left(\psi \right)$ and $\omega_{2}\left(\psi \right)$ as follows:

(1) $$\rho ={\omega_{1}\left(\psi \right)}^{T}⊗\omega_{2}\left(\psi \right),$$

where ⊗ denotes the matrix multiplication and ω(.) is a 1×1 convolution. The self-attention mechanism follows the concept of the non-local means filtering technique [41,42]. The non-local means technique computes a weighted mean of all the pixels in an image and allows distant pixels to contribute to the filtered response at a location based on the patch similarity [43]. Notably, the self-attention mechanism of previous studies [41,42] calculates feature dependencies at the global aspect, as performed in the non-local means. The self-attention method uses the matrix multiplication in the feature level to calculate feature dependencies, as stated in Equation (1). The global correlation map obtained from this multiplication contains correlation values among all possible pixel pairs of the Feature 1 and Feature 2, as shown in Figure 3.

We then perform the mask pruning by multiplying mask *M*. By multiplying with the mask *M*, we remove the less important correlation features and only concentrate on the features that contribute to better inpainting information. The pruned correlation map is given by

(2) $$\rho_{M}=\rho ⊙\;M,$$

where ⊙ denotes element-wise multiplication.

After obtaining the pruned correlation map, a softmax operation is performed to achieve the final global attention score $\kappa_{\rho }$.

(3) $$\kappa_{\rho }=\frac{\exp(\rho_{M})}{\sum_{i}\exp\left(\rho_{M}^{i}\right)}.$$

We obtain the attended features $\psi_{p}$ by multiplying the attention score $\kappa_{\rho }$ with $\omega_{3}\left(\psi \right)$. The final output $\psi_{out}$ is obtained by replacing the attended features $\psi_{p}$ with the mask region *M*.

(4) $$\psi_{p}=\omega_{3}\left(\psi \right)⊗{\left(\kappa_{\rho }\right)}^{T}.$$

(5) $$\psi_{out}=M\times \psi +\left(1-M\right)times\psi_{p}.$$

### 3.2. Refinement Network

The MPGA module in the coarse network can capture the global semantic relationship among the features and can effectively prune less important features to achieve a better estimation of the missing regions. Because the refinement network is responsible for the refined output, along with the global dependency for structural consistency, it is also essential to capture the local similarities among the features for a consistent texture. Although the existing models focus on a local similarity using contextual information based on the inner product [14,16] or inter-layer self-attention mechanism [39], we propose that it is logical to prune out the features in global terms, and use the pruned features to calculate the local similarity. We propose a novel GLA module that can efficiently prune the global features and calculate the local similarity based on the pruned patches extracted from the pruned features in local terms. Section 3.2.1 provides an overview of the refinement network architecture and Section 3.2.2 describes the proposed GLA module.

#### 3.2.1. Refinement Network Architecture

The refinement network is also an encoder-decoder network with two separate branches, regular and attention branches, in the encoder segment. In the case of a coarse network, the initial input $I_{in}$ is an image consisting of holes. However, in the case of a refinement network, the coarse estimation output $I_{coarse}$ from the coarse network is given as an input to the refinement network. Specifically, the coarse output image $I_{coarse}$ and the mask *M* are concatenated channel-wise and fed into both the regular and attention branches of the refinement network. Because a refinement network deals with more information compared with a coarse network, we integrate the proposed GLA module in the attention branch of the refinement network to have a refined output.

As with the coarse network, in a regular branch, we use 3×3 convolution layers with dilated convolutions and kernel sizes of 3×3 with rates of 2, 4, 8, and 16 to achieve a better feature extraction. In the attention branch, we down-sample the feature map with a convolution operation with a kernel size of 3×3 and stride 2. We then integrate our proposed module to provide both global and local views of the semantic relationship among features and obtain a pruned feature map. The pruned feature map along with a raw feature map is then used to find a local similarity for feature map reconstruction. The outputs from both regular and attention branches are concatenated and fed into a single decoder that outputs the refined image $I_{rec}$. The final inpainted result $I_{inpainted}$ is obtained by pasting the masked region of the refined image into the input image $I_{in}$, i.e., $I_{inpainted}=I_{in}+I_{rec}⊙\left(1-M\right)$ (see Appendix B.2 for the refinement network architecture).

#### 3.2.2. Global and Local Attention Module

Most of the existing studies [16,17,19] have focused on local similarities only, whereas other studies [39] have focused on inter-intra global information only for the refinement of the results. However, depending on the local similarities for the refinement has certain limitations. First, only patch-based local similarities can generate repetitive and ambiguous content. Second, discontinuities among the missing and background regions can be visible owing to a lack of global semantic information.

**Figure 4:**
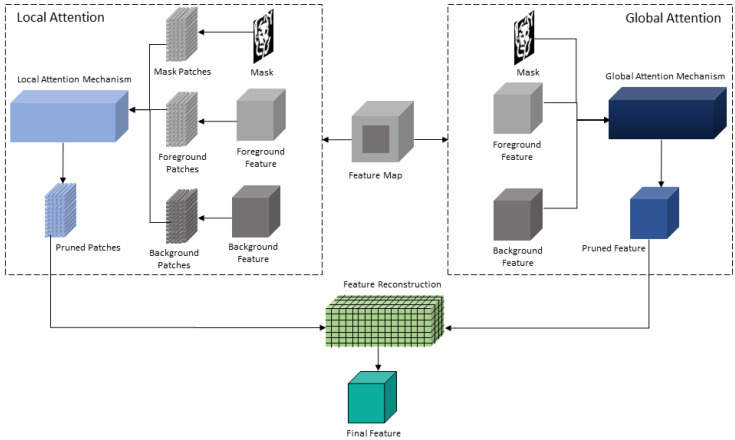
Overview of the proposed GLA module. Our proposed module takes the foreground and background feature maps and calculates both the global attention and local similarity for a refinement of the coarse output. It then calculates the pruned feature from the global attention mechanism and pruned patches from the local attention mechanisms for calculating the local similarity based on the inner product. It then uses the pruned patches to reconstruct the final feature map.

Our proposed GLA module takes feature maps and masks as input. First, the module divides the feature maps into the foreground and background features. Patches are then extracted from the features and the mask to generate multiple candidate patches for a similarity calculation. The module then calculates global and local similarities among the patches and selects the best patches for reconstructing the features. Figure 4 shows an abstract view of the workflow of GLA module.

Formally, the input feature ψ is divided into a foreground feature ($\psi_{f}$) and a background feature ($\psi_{b}$). The module then extracts foreground patches ($p_{\psi_{f}}$), background patches ($p_{\psi_{b}}$), and mask patches ($p_{M}$) for mask-pruning at the patch-level. Along with extracting the patches, the module first applies the global attention mechanism along with mask-pruning using Equation (5). Then, the module calculates the patch-level correlation $\rho_{p}$ and performs channel-wise mask pruning to obtain $\rho_{p_{M}}$.

(6) $$\rho_{p}={\omega_{1}\left(p_{\psi_{f}}\right)}^{T}⊗\omega_{2}\left(p_{\psi_{b}}\right),$$

(7) $$\rho_{p_{M}}=\rho_{p}⊙p_{M},$$

where ω(.) is a 1×1 convolution.

The local attention score is then given by a softmax operation as follows:

(8) $$\kappa_{\rho_{p}}=\frac{\exp(\rho_{p_{M}})}{\sum_{i}\exp\left(\rho_{p_{M}}^{i}\right)}.$$

Then, based on the attention scores $\kappa_{\rho_{p}}$ of the patches, the module calculates the pruned patches $p_{\psi_{p}}^{{}^{\prime }}$ as follows:

(9) $$p_{\psi_{p}}=\omega_{3}\left(p_{\psi_{f}}\right)⊗{\left(\kappa_{\rho_{p}}\right)}^{T},$$

(10) $${p^{{}^{\prime }}}_{\psi_{p}}=p_{M}\times p_{\psi_{f}}+\left(1-p_{M}\right)\times p_{\psi_{p}}.$$

After obtaining the pruned patch $p_{\psi_{p}}^{{}^{\prime }}$, the module calculates the local similarity based on the inner product from the pruned feature map $\psi_{out}$ using the pruned patches $p_{\psi_{p}}^{{}^{\prime }}$ as convolution filters. It has been shown that using patches as convolution filter kernels can effectively calculate the local similarity, as mentioned in previous studies [16]. The local similarity between the pruned path $p_{\psi_{p}}^{{}^{\prime }}$ and the $i^{th}$ patch of the pruned feature map (i.e., $p_{\psi_{out}}^{i}$) is given by the following:

(11) $$s_{p_{\psi_{p}}^{{}^{\prime }},p_{\psi_{out}}}^{i}=<\frac{{p^{{}^{\prime }}}_{\psi_{p}}}{\left|\right|p_{\psi_{p}}^{{}^{\prime }}\left|\right|},\frac{p_{\psi_{out}}^{i}}{\left|\right|p_{\psi_{out}}^{i}\left|\right|}>.$$

The final attention score is then computed based on the local similarity s as follows:

(12) $$\kappa_{s}=\frac{\exp(s)}{\sum_{i}\exp\left(s^{i}\right)}.$$

The module then multiplies the pruned patch $p_{\psi_{p}}^{{}^{\prime }}$ with the final attention score $\kappa_{s}$ and selects the patches with the higher attention values. Finally, a deconvolution is performed to reconstruct the final feature map using the most contributing patches as the convolution filters. Although the mask pruning mechanism is similar to the proposed MPGA module, the MPGA module calculates the global dependency information from the features only, whereas the GLA module divides the feature maps into both feature maps and patches and simultaneously calculates both the global dependencies and the local similarities.

### 3.3. Discriminator

**Figure 5:**
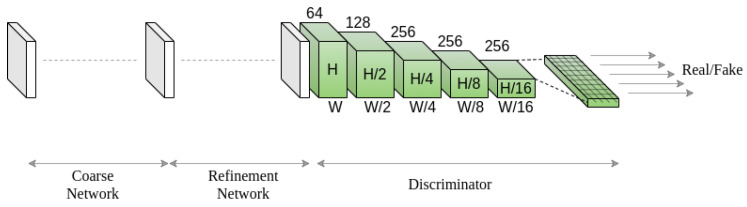
Overview of the discriminator network.

In the case of free-form image inpainting, masks can appear anywhere in the image with any shape. Thus, incorporating a global discriminator for global-level supervision is ineffective. Hence, we designed a discriminator based on the spectral normalization [44] and relativistic average hinge GAN [45], which can act as a local discriminator while evaluating different locations of the feature map. Figure 5 shows an overview of the discriminator (see Appendix B.3 for details on the discriminator network architecture).

We designed a convolutional neural network-based discriminator that takes the inpainted image and respective ground truth as inputs and generates two feature volumes of shape $R^{c\times h\times w}$ where *c*, *h* and *w* represent channels, height and width, respectively. The discriminator consists of five convolutional layers with 5×5 kernels with a stride of 2 to capture the feature statistics [46]. We apply GANs for each element in the final feature volume generated by the discriminator [16], as shown in Figure 5. The discriminator calculates the probabilities whether the features in the generated feature volume are real or fake, with respect to the features in the ground truth feature volume. Using the CNN-based discriminator, the receptive fields of each point in the output map can cover the input image, as shown in Figure 5. A dropout layer is incorporated in the discriminator to allow stochasticity in the model.

### 3.4. Objective Function

For the coarse reconstruction, we adopt a weighted $L_{1}+SSIM$ loss, where $L_{1}$ calculates the absolute difference of the values, and SSIM calculates the luminance, contrast, and structural similarity of the images [47,48]. The reconstruction function, $L_{recon}$, is given by the following:

(13) $$L_{recon}=\lambda \times L_{L_{1}}+\left(1-\lambda \right)\times L_{SSIM},$$

where λ is the weighting factor between $L_{1}$ and SSIM and accumulates at 1.

For adversarial guidance, we adopt the relativistic average hinge loss [45]. The adversarial setup can be defined as follows:

(14) $$L_{hinge}^{D}=E_{x_{r}}\;P\left[\max\left(0,1-\tilde{D}\left(x_{r}\right)\right)\right]+E_{x_{f}}\;Q\left[\max\left(0,1+\tilde{D}\left(x_{f}\right)\right)\right],$$

(15) $$L_{hinge}^{G}=E_{x_{f}}\;P\left[\max\left(0,1-\tilde{D}\left(x_{f}\right)\right)\right]+E_{x_{r}}\;Q\left[\max\left(0,1+\tilde{D}\left(x_{r}\right)\right)\right],$$

Here, $\tilde{D}\left(x_{r}\right)$ and $\tilde{D}\left(x_{f}\right)$ are defined as follows:

(16) $$\tilde{D}\left(x_{r}\right)=C\left(x_{r}\right)-E_{x_{f}}\;Q\left[C\left(x_{f}\right)\right],$$

(17) $$\tilde{D}\left(x_{f}\right)=C\left(x_{f}\right)-E_{x_{r}}\;P\left[C\left(x_{r}\right)\right],$$

where C(.), *P*, *Q*, $x_{r}$, and $x_{f}$ are the non-transformed discriminator output, distribution of real data, distribution of generated data, real data, and generated data, respectively. In addition, C(.) can be interpreted as indicating how realistic the input data are compared with the generated data [45,49]. Moreover, $L_{hinge}^{D}$ and $L_{hinge}^{G}$ are the relativistic average hinge discriminator loss and the relativistic average hinge generator loss, respectively. We also adopt a loss called the Lorentzian loss, $L_{loren}$ [50], which calculates the absolute logarithmic distances between values.

(18) $$L_{loren}=\log\left(1+|P-Q|\right),$$

where *P* and *Q* are the distributions of the real data and generated data, respectively. We use the Lorentzian loss for both the generator and discriminator and denote them as $L_{loren}^{G}$ and $L_{loren}^{D}$, respectively.

Due to $L_{L_{1}}$ and SSIM, the model tries to minimize the per-pixel differences with smaller penalties during image reconstruction, whereas the Lorentzian loss has a higher penalty when evaluating the discrimination. Hence, the model tries to generate novel contents with minimum differences with the ground truth during training and generates more plausible and consistent results during testing. The overall objective functions for the generator and discriminator, $L^{G}$ and $L^{D}$, can be defined as follows:

(19) $$L^{G}=L_{recon}+L_{hinge}^{G}+L_{loren}^{G}.$$

(20) $$L^{D}=L_{hinge}^{D}+L_{loren}^{D}.$$

## 4. Experimental Results

We evaluate our proposed method on three popular datasets: Places365 [21], ImageNet [22], and CelebA-HQ [23]. We use the original training, testing, and validation splits for Places365 and ImageNet. For CelebA-HQ, we use the last 3000 images as the testing images and the rest for training because CelebA-HQ does not have a predefined training-testing split. We compared our proposed method with seven existing state-of-the-art methods: contextual attention (CA) [14], partial convolution (PC) [17], generative multicolumn (MC) [18], gated convolution (GC) [16], pluralistic (PL) [39], EdgeConnect (EC) [19] and DeepFusion (DF) [40]. We refer to official implementations along with the pre-trained weights from the respective authors and conduct an evaluation without any major modifications of the original setups. Because the PL method [39] does not provide any test codes for evaluating a single image (at the time of the evaluation), we choose the most visually plausible results from the inpainted images. For comparisons in ImageNet and CelebA-HQ datasets, we excluded some methods due to the unavailability of pre-trained models for the datasets (i.e., the DF method for ImageNet and PC method for CelebA-HQ). Note that [20] requires additional structural images in the testing phase, and we exclude this method because all other methods use only input images and masks for this phase.

Our model is optimized using the Adam algorithm with a learning rate of $\alpha =1\times 10^{-4}$ and we set $\beta_{1}$ and $\beta_{2}$ as 0.5 and 0.9. We train our model on a single NVIDIA TITAN XP GPU with a batch size of 4. To generate the free-form masks for training and testing, we follow the GC method [16], which allows an on-the-fly mask generation instead of the predefined masks used in the PC method [17]. The sizes of the test images for the Places365 and ImaageNet datasets are 512×680 and 256×256 for CelebA-HQ.

### 4.1. Qualitative Results

**Figure 6:**
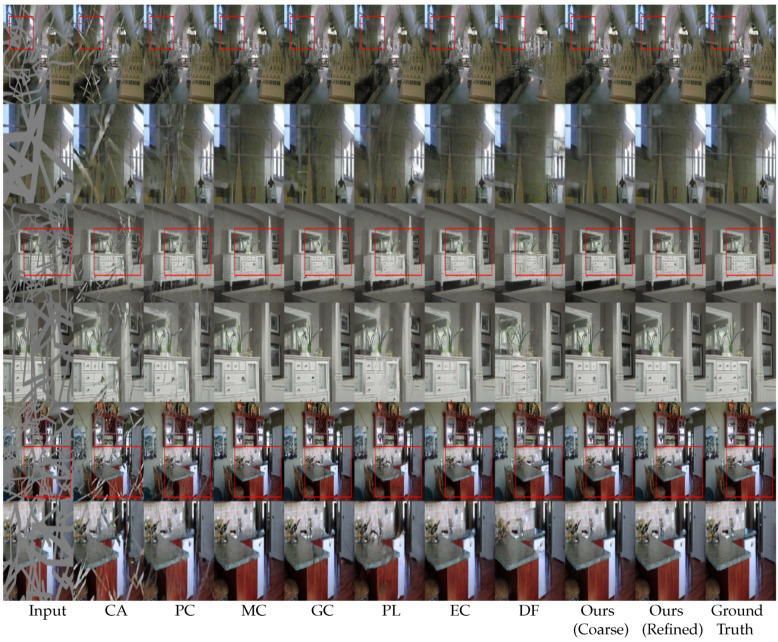
Qualitative comparison of Places365 [21] dataset. From the left: input, CA [14], PC [17], MC [18], GC [16], PL [39], EC [19], DF [40], proposed method (coarse output), proposed method (refined output), and the ground truth. It can be seen from the image that our model generates more consistent structures and textures compared with the state-of-the-art methods.

**Figure 7:**
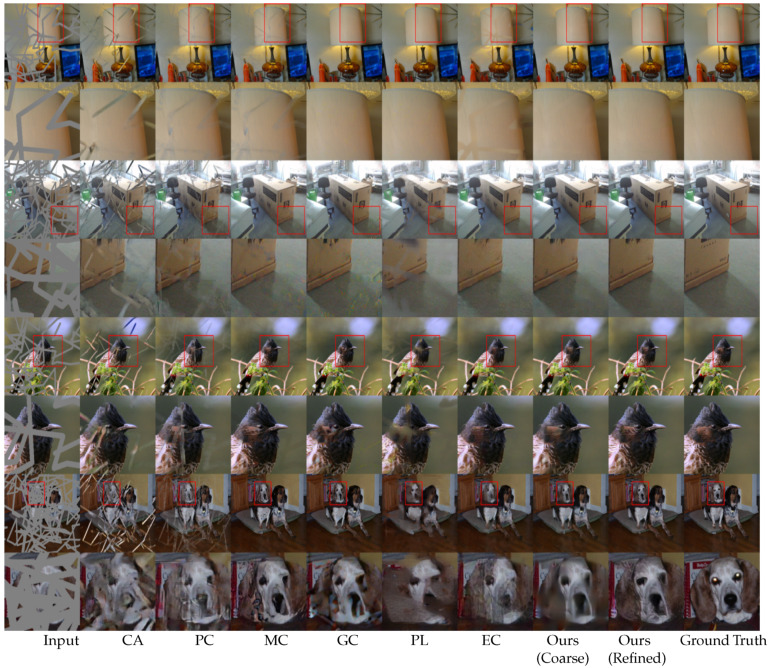
Qualitative comparison on ImageNet [22] dataset. From the left: input, CA [14], PC [17], MC [18], GC [16], PL [39], EC [19], proposed method (coarse output), proposed method (refined output), and the ground truth. Our model generates realistic and consistent structures and textures for larger holes.

**Figure 8:**
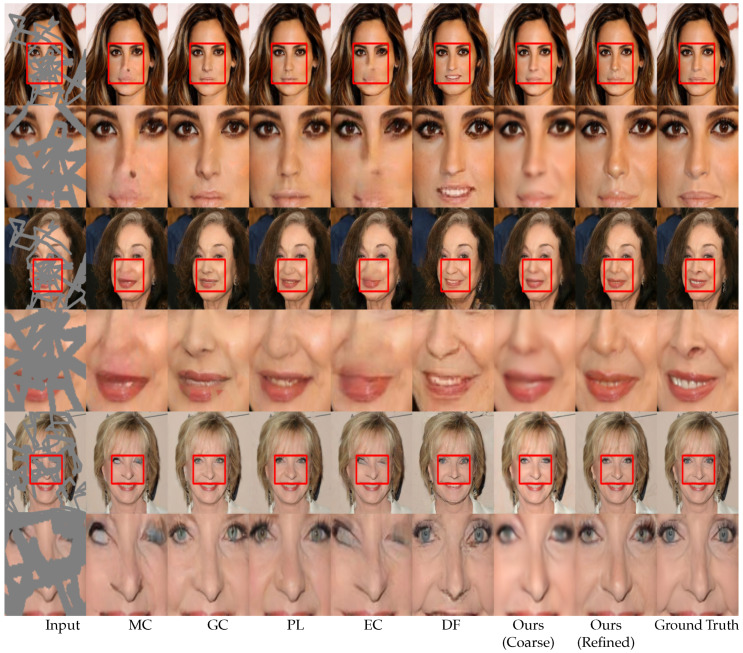
Qualitative comparison on CelebA-HQ [23] dataset. From the left: input, MC [18], GC [16], PL [39], EC [19], DF [40], proposed method (coarse output), proposed method (refined output), and the ground truth.

For a qualitative evaluation, we provided visual comparisons in Figure 6 for Places365 consisting of complex natural scenes, Figure 7 for the ImageNet dataset consisting of indoor scenes, and Figure 8 for the CelebA-HQ dataset consisting of human faces. Each figure consists of comparison images and zoomed-in views of the images for better visualization.

As indicated in the figures, the CA method [14] generates ambiguous contents due to a lack of an adaptive mask update mechanism. Although the PC method [17] focuses on a mask update mechanism, it fails when the free-form mask becomes larger in size and width. The mask update mechanism of the PC method [17] is rule-based or heuristic, which classifies all spatial pixel locations to be either valid or invalid based on predefined rule (i.e., 1 for valid pixel and 0 for invalid pixel). This update mechanism often fails to update the correct mask values due to the heuristic rules, as mentioned in previous studies [16]. The MC method [18] performs better in texture inpainting owing to the multi-scale feature extraction mechanism. However, it performs poorly at the edges and in the consistent blending of the structures. The GC method [16] generates plausible results in repetitive structures due to the patch-based inner product similarity mechanism and soft-gating. However, it fails in plain textures and tends to produce ’tails’ (i.e., repetitive structures) or inconsistent contents (white spots). The PL method [39] can generate multiple inpainting candidates due to the variational approach. However, it performs poorly in terms of both texture and structure in complex natural scenes as neighboring pixels in natural scenes are generally similar, which the PL method [39] does not explicitly consider. The EC method [19] can generate plausible structures due to the edge hallucination mechanism. However, the EC method [19] generates inconsistent structures and textures due to a poor edge prediction from the use of the Canny edge detection mechanism [51], which is sensitive to noise and thresholding. As a result, the EC [19] performs poorly in plain textures. The DF method [40] uses fusion blocks to efficiently blend the inpainting results into the background. However, the merging operation in the DF method [40] is inefficient and leads to inconsistent content in the generated images.

Our proposed model uses MPGA module in the coarse network that provides additional structural information to the model. Hence, our model can hallucinate structures without any additional guidance, such as an EC method [19]. As seen in Figure 6, Figure 7 and Figure 8, the coarse network of our proposed model hallucinates structurally consistent yet blurry coarse outputs. Then, the refinement network takes the coarse outputs as inputs and performs local and global level refinement. The GLA module in the refinement network prunes the features at the global level and calculates the similarity at the local level, which provides additional guidance for the refinement of the coarse estimation (see Appendix A for more visual results).

### 4.2. Quantitative Results

Evaluation of image inpainting is comparatively subjective as deep learning models generate novel contents for missing regions, and hence traditional evaluation metrics such as $L_{1}$ error and SSIM values do not justify the effectiveness of the models [14]. However, for a quantitative evaluation, we choose four popular evaluation metrics, i.e., $L_{1}$ error, $L_{2}$ error, SSIM, and PSNR, which are generally used in the field of inpainting. We also choose 1000 random images from the Places365 validation set and ImageNet test set. For a CelebA comparison, we use the last 3000 images as the test images, while using the rest of the images as the training data. Table 1, Table 2 and Table 3 show a quantitative evaluation of the proposed method and the comparison models in the Places365, ImageNet, and CelebA-HQ datasets, respectively.

Because the Places365 and ImageNet datasets consist of complex natural and indoor scenes, respectively, inpainting models need to capture the underlying structures and semantic relationships of the features. Hence, the proposed global attention module captures the global dependencies of the features. Moreover, it is important to achieve texture consistency among the features to have a better blending of the inpainting results and background. Our proposed GLA module extracts similar patches from the background, prunes the patches, and then uses the pruned patches to reconstruct the background. As a result, the inpainting results are more realistic and consistent with the surrounding background. The CelebA-HQ dataset consists of human faces that require texture consistency for generated images. Our proposed model can handle global structure consistency and local texture consistency owing to the proposed MPGA module and the GLA module.

From Table 1, Table 2 and Table 3, it can be seen that our proposed model achieves comparatively lower values in terms of the $L_{1}$ and $L_{2}$ errors, while attaining higher SSIM and PSNR values even for larger holes. It can be seen that the CA method [14] has larger $L_{1}$ and $L_{2}$ errors as well as a lower SSIM and PSNR because [14] depends on only local similarities among patches and cannot handle free-form holes. The PC method [17] updates the mask heuristically and cannot capture the semantic dependencies of the features. The MC method [18] shows a comparatively higher SSIM and PSNR because it depends on multi-scale feature extraction and regularization. However, although the MC method [18] shows comparative results, it cannot handle larger free-form holes due to a lack of a mask update mechanism. The GC method [16] can handle larger holes and produces better-inpainting results owing to the soft-gating mechanism for a mask update. However, because it relies only on the inner product similarity, it tends to produce repetitive results and artifacts. Our proposed model incorporates mask pruning in both global features and local patches, which can effectively prune out the mask features from the global dependencies and can calculate both global and local similarities for better texture and structural consistency, leading to better-inpainting results compared with the existing methods.

Table 4 shows the comparison of the number of trainable parameters and model inference time. The proposed model has 8.71 M trainable parameters, which is less than the existing methods. However, the inference time of the proposed model is slower (0.21 sec) than that of the existing methods (e.g., 0.12 s for the PC method [17]) as the proposed model calculates both global and local level similarities, which leads to comparatively slower inference. The comparison was done in a single NVIDIA Titan XP GPU with PyTorch 1.1 for the PL method [39], the EC method [19], the DF method [40] and the proposed model. For the CA method [14], the GC method [16] and the MC method [18], TensorFlow 1.15 was used. For tensor processing, CUDNN v7.0 and CUDA v10.0 were used. For the comparison, test image and mask size were 512×512.

### 4.3. Ablation Study

To evaluate our proposed MPGA module, GLA module, and Lorentzian loss, we conducted ablation studies by replacing the modules with convolution layers and removing the loss. We consider two cases, namely without the MPGA module and without the GLA module.

**Figure 9:**
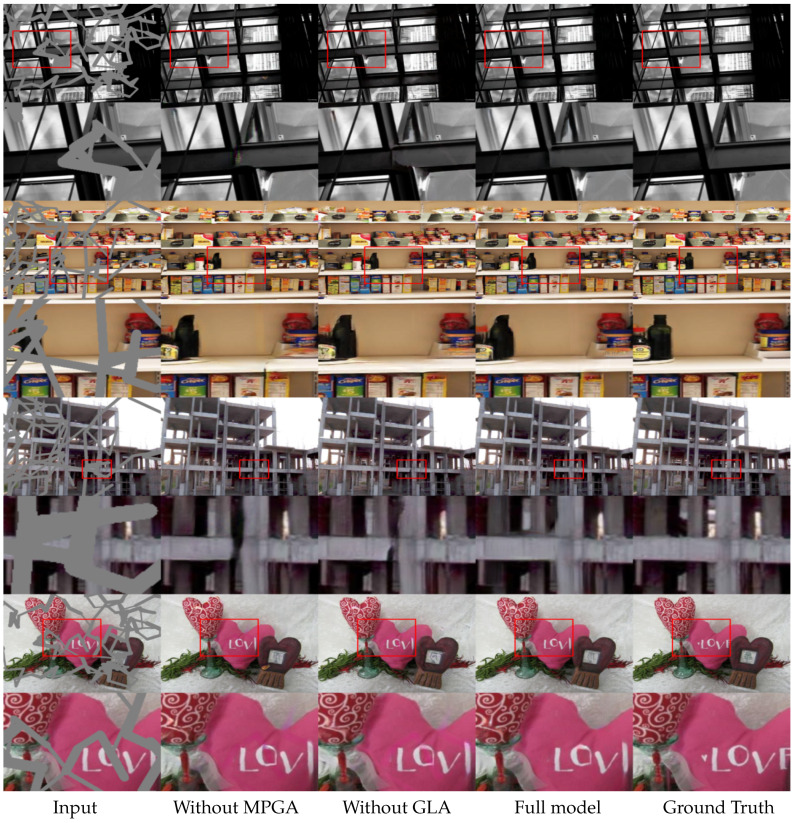
Ablation study of the proposed modules in the Places365 dataset [21]. From left: input image, without the MPGA module, without the GLA module, full model with the proposed modules, and the ground truth.

To visualize the effectiveness of the proposed MPGA module in the coarse network, we conducted ablation studies without the proposed module. In this study, the proposed module was replaced with a 3×3 convolution layer and with all other structures being the same. It can be seen from Figure 9 that, without the proposed module, the network generates blurry textures and less consistent structures. To understand the effects of the proposed GLA module, we replaced the module with a 3×3 convolution and trained the model. It can be seen from Figure 9 that, without the proposed module, the model tends to generate less consistent in both texture and structure.

From Table 5, it can be seen that although the MPGA mechanism in the first model can provide global dependency information, it lacks the local information needed for a refinement of the inpainting results. Moreover, because there are no global or local attention mechanisms in the first model, the model produces structurally consistent yet unrefined results. The second model consists of only a GLA mechanism and performs better than the first model consisting of only the MPGA mechanism. Due to the availability of both global and local information, the second model generates comparatively better results. However, as the refinement network depends on the coarse output, and the second model lacks a global attention mechanism in the coarse network, it produces a better texture transition while lacking structural consistency. The full model consists of both proposed modules and generates visually plausible and consistent inpainted images.

**Figure 10:**
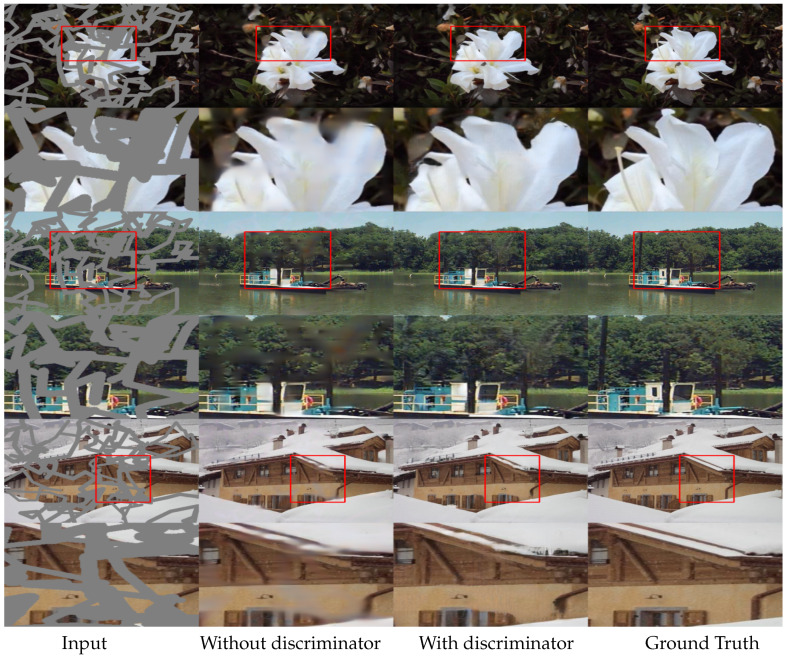
Ablation study for the effect of the discriminator in the proposed model in Places365 dataset [21]. From left: input image, output (without discriminator), output (with discriminator), and the ground truth.

Figure 10 shows the effects of the discriminator for the inpainted results. For the comparison, the proposed model has been trained with only the reconstruction loss $L_{recon}$ (i.e., Equation (13)). Owing to the MPGA and GLA, the model without the discriminator performs both global and local level refinement and can capture the essence of the missing region. However, it still fails to generate visually plausible results with realistic details. As the discriminator provides supervision on content generation for the missing region, the absence of the discriminator will inevitably produce blurry output. As seen in Figure 10, our proposed model with the discriminator, combined with the MPGA and GLA, can produce visually plausible and realistic details for the missing regions.

## 5. Conclusions

We proposed an image inpainting architecture with two novel attention modules for deep learning-based image inpainting, namely a mask pruning-based global attention module for a coarse estimation of missing regions and a global and local attention module for the refinement of the generated contents. Our method generates a structurally consistent coarse estimation of the missing regions based on the mask pruning mechanism proposed in the mask pruning-based global attention module integrated into the coarse network and can generate fine details owing to the global and local similarity calculations based on the proposed global and local attention module integrated into the refinement network. From the quantitative and qualitative evaluations, it can be seen that our proposed method can generate structurally consistent contents along with a good texture without any pre-processing, and outperforms existing methods in terms of the $L_{1}$ error, $L_{2}$ error, SSIM error, and PSNR in most cases. The future direction of this research is to find new similarity measures and new attention mechanisms to generate very high-resolution contents for image inpainting tasks.

## Figures and Tables

**Table 1:** Quantitative comparison on Places365 [21] dataset.

Metrics/Models	Mask Ratio	CA [14]	PC [17]	MC [18]	GC [16]	PL [39]	EC [19]	DF [40]	Ours
$L_{1}$	20–30%	3.50	2.60	1.61	1.41	2.20	1.90	2.63	**1.20**
	30–40%	4.11	3.15	2.48	1.62	2.63	2.50	2.88	**1.44**
	40–50%	5.69	4.41	2.94	2.39	2.98	3.01	3.15	**2.23**
$L_{2}$	20–30%	0.97	0.33	0.26	0.23	0.34	0.24	0.41	**0.20**
	30–40%	1.15	0.39	0.43	0.25	0.41	0.26	0.56	**0.21**
	40–50%	1.55	0.57	0.56	0.45	0.47	0.48	0.61	**0.36**
SSIM	20–30%	81.32	83.22	89.16	90.11	85.21	86.61	83.16	**92.91**
	30–40%	69.77	78.84	82.45	88.26	82.64	84.57	80.19	**89.54**
	40–50%	67.31	71.63	79.50	82.01	78.87	78.29	76.98	**84.39**
PSNR	20–30%	21.25	25.56	26.99	28.16	27.62	27.77	26.84	**28.89**
	30–40%	20.45	24.81	24.73	27.82	27.25	27.56	25.43	**28.67**
	40–50%	19.03	23.29	23.49	25.75	25.47	24.57	24.01	**26.11**

**Table 2:** Quantitative comparison on ImageNet dataset [22].

Metrics/Models	Mask Ratio	CA [14]	PC [17]	MC [18]	GC [16]	PL [39]	EC [19]	Ours
$L_{1}$	20–30%	3.96	2.91	2.23	2.30	2.78	2.67	**2.01**
	30–40%	4.95	3.66	2.99	3.14	3.55	3.42	**2.78**
	40–50%	6.63	4.47	3.38	3.57	3.74	3.82	**3.21**
$L_{2}$	20–30%	1.36	0.38	0.29	0.32	0.36	0.32	**0.26**
	30–40%	1.70	0.51	0.45	0.51	0.58	0.56	**0.43**
	40–50%	2.34	0.66	0.56	0.64	0.69	0.61	**0.55**
SSIM	20–30%	79.73	81.06	**88.38**	87.94	84.13	82.03	87.58
	30–40%	73.60	75.65	81.95	81.17	79.95	75.67	**82.04**
	40–50%	66.46	70.88	78.16	78.31	76.23	73.05	**79.14**
PSNR	20–30%	19.77	24.76	26.95	26.45	26.88	26.16	**27.15**
	30–40%	18.87	23.46	24.87	24.26	24.01	24.19	**25.03**
	40–50%	17.47	22.35	23.61	22.96	22.67	23.04	**23.88**

**Table 3:** Quantitative comparison on CelebA-HQ dataset [23].

Metrics/Models	Mask Ratio	MC [18]	GC [16]	PL [39]	EC [19]	DF [40]	Ours
$L_{1}$	20–30%	1.85	**1.49**	1.87	2.29	2.37	1.55
	30–40%	2.18	2.43	2.52	2.39	3.39	**1.73**
	40–50%	2.37	2.55	2.38	2.93	4.17	**2.25**
$L_{2}$	20–30%	0.21	0.20	0.25	0.22	0.93	**0.18**
	30–40%	0.33	0.32	0.29	0.24	0.99	**0.21**
	40–50%	0.38	0.33	0.35	0.37	1.51	**0.32**
SSIM	20–30%	89.66	89.42	89.64	85.47	85.56	**90.77**
	30–40%	86.24	86.06	86.13	84.30	82.12	**89.01**
	40–50%	85.14	84.63	84.76	80.78	79.46	**85.69**
PSNR	20–30%	27.64	**28.13**	27.59	27.05	25.62	28.05
	30–40%	25.48	25.40	26.88	26.76	23.74	**27.53**
	40–50%	25.11	25.34	24.49	24.74	22.58	**25.45**

**Table 4:** Comparison of the number of trainable parameters and model inference time.

Model	Parameters (M)	Inference Time (s)
CA	12.8	0.22
PC	85.75	**0.12**
MC	15.07	0.14
GC	10.0	0.18
PL	18.42	0.18
EC	27.15	0.15
DF	32.89	0.15
**Proposed**	**8.71**	0.21

**Table 5:** Ablation study of the proposed modules in Places365 dataset [21].

Metrics/Modes	${\mathrm{MPGA}}^{+}+{\mathrm{GLA}}^{-}$	${\mathrm{MPGA}}^{-}+{\mathrm{GLA}}^{+}$	Full
L1	1.61	1.54	1.28
L2	0.26	0.24	0.22
SSIM	90.57	90.76	91.42
PSNR	27.02	27.22	27.70

Here, $MPGA^{+}$ and $MPGA^{-}$ denote the methods with and without the MPGA module in the coarse network, and $GLA^{+}$ and $GLA^{-}$ denote the methods with and without the global and local attention module in the refinement network.

**Figure A1:**
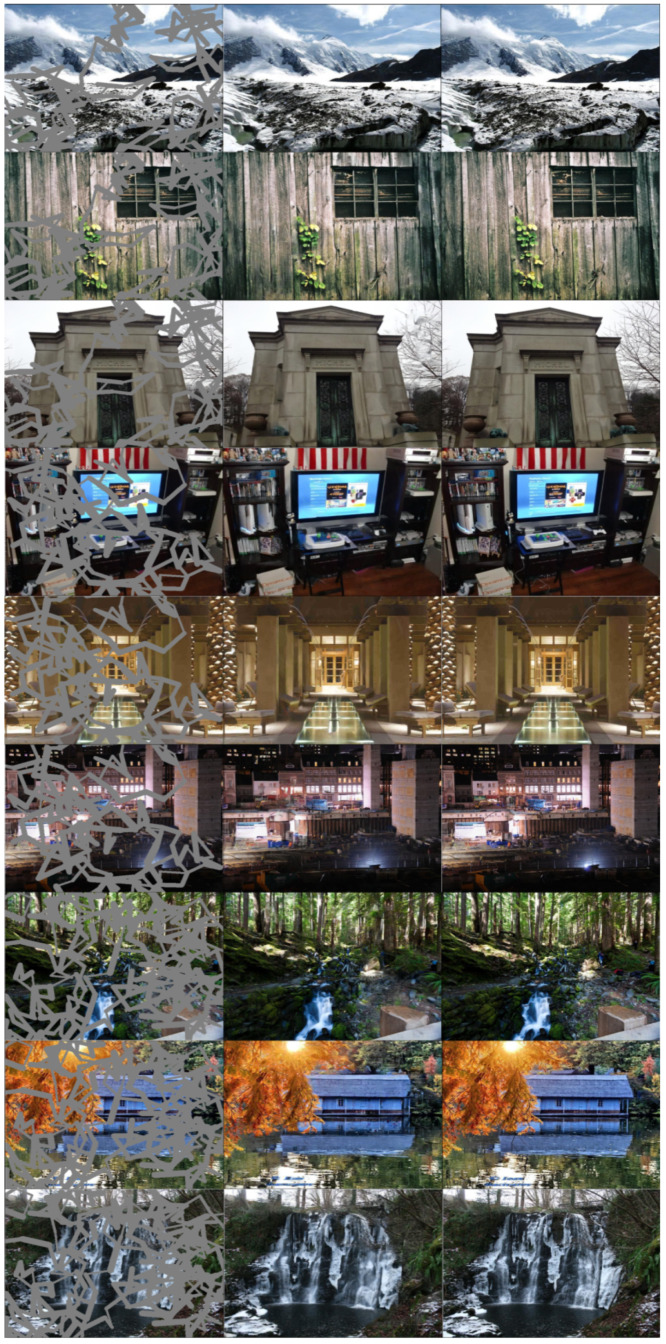
More results on Places365 dataset [21]. From left: input image, inpainted image, and respective ground truth.

**Figure A2:**
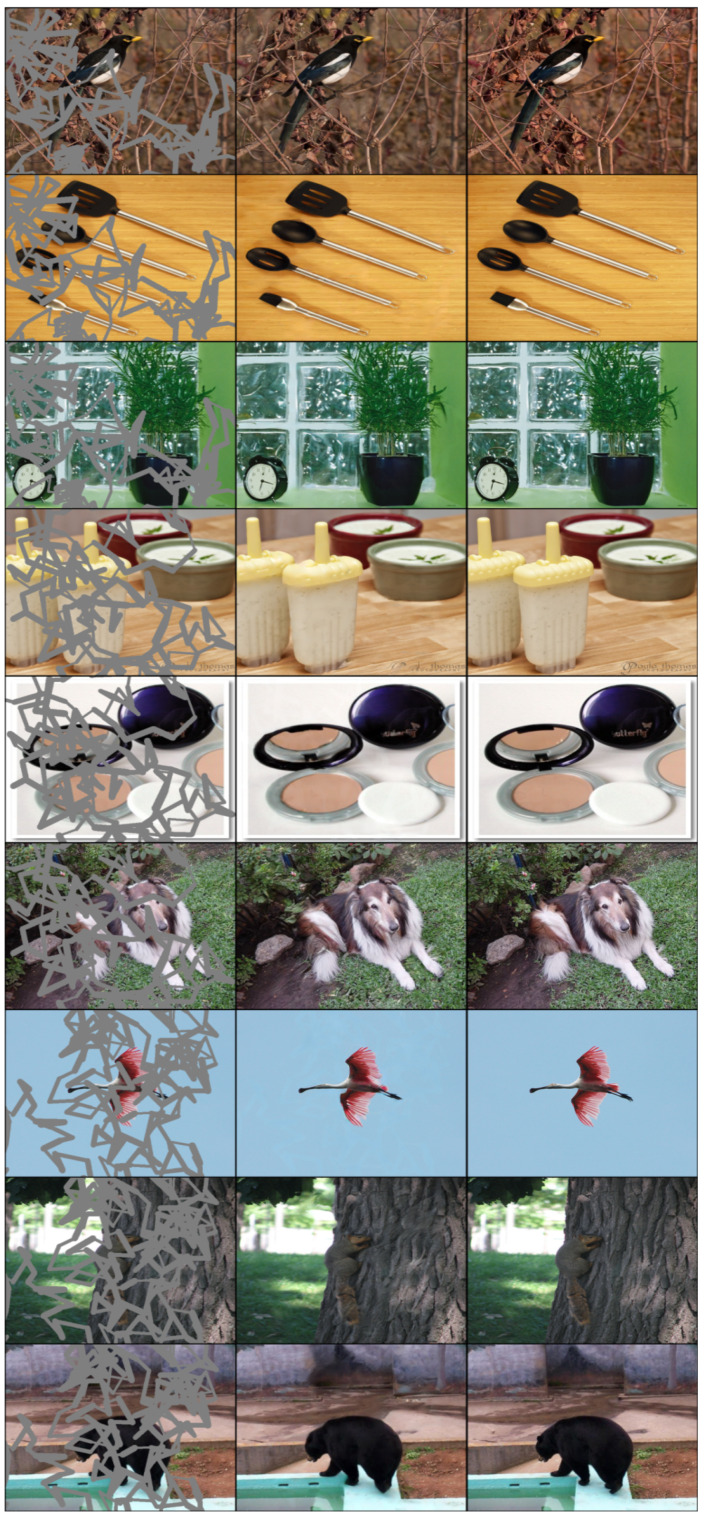
More results on ImageNet dataset [22]. From left: input image, inpainted image, and respective ground truth.

**Figure A3:**
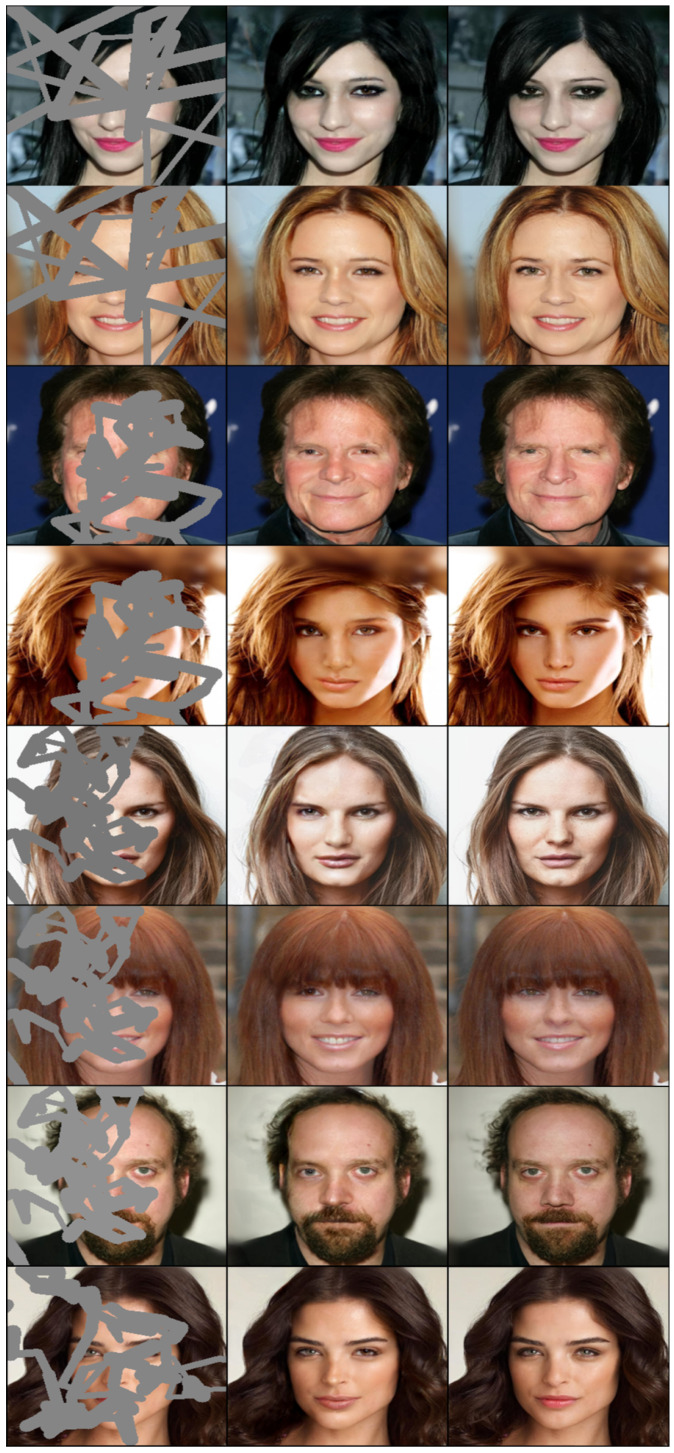
More results on CelebA-HQ dataset [23]. From left: input image, inpainted image, and respective ground truth.

## Appendix B. Network Architectures

### Appendix B.1. Coarse Network Architecture

**Table A1:** Coarse Network Architecture.

Inputs/Branches	Operation	Kernel Size	Dilation	Stride	Output	Activation
Input	Concatenation (Input Image, Mask)					
Regular Branch	Convolution	5 × 5	1	1	32	Leaky ReLU
Regular Branch	Convolution	3 × 3	1	2	32	Leaky ReLU
Regular Branch	Convolution	3 × 3	1	1	64	Leaky ReLU
Regular Branch	Convolution	3 × 3	1	2	64	Leaky ReLU
Regular Branch	Convolution	3 × 3	1	2	128	Leaky ReLU
Regular Branch	Convolution	3 × 3	1	1	128	Leaky ReLU
Regular Branch	Atrous Convolution	3 × 3	2	1	128	Leaky ReLU
Regular Branch	Atrous Convolution	3 × 3	4	1	128	Leaky ReLU
Regular Branch	Atrous Convolution	3 × 3	8	1	128	Leaky ReLU
Regular Branch Output	Atrous Convolution	3 × 3	16	1	128	Leaky ReLU
Attention Branch Input	Concatenation (Input Image, Mask)					
Attention Branch	Convolution	5 × 5	1	1	32	Leaky ReLU
Attention Branch	Convolution	3 × 3	1	2	32	Leaky ReLU
Attention Branch	Convolution	3 × 3	1	2	64	Leaky ReLU
Attention Branch	Convolution	3 × 3	1	2	128	Leaky ReLU
Attention Branch	Convolution	3 × 3	1	2	128	Leaky ReLU
MPGA Module	Attention	3 × 3	2	1	128	None
Attention Branch	Convolution	3 × 3	1	1	128	Leaky ReLU
Attention Branch Output	Convolution	3 × 3	1	1	128	Leaky ReLU
Combined Layer	Concatenation (Regular Branch Output, Attention Branch Output)					
Combined Layer	Convolution	3 × 3	1	1	128	Leaky ReLU
Combined Layer	Convolution	3 × 3	1	1	128	Leaky ReLU
Combined Layer	Upscale	Factor of 2				None
Combined Layer	Convolution	3 × 3	1	1	64	Leaky ReLU
Combined Layer	Convolution	3 × 3	1	1	64	Leaky ReLU
Combined Layer	Upscale	Factor of 2				None
Combined Layer	Convolution	3 × 3	1	1	32	Leaky ReLU
Combined Layer	Convolution	3 × 3	1	1	16	Leaky ReLU
Combined Layer	Convolution	3 × 3	1	1	3	Leaky ReLU
Coarse Inpainted Image	Clamp [−1,1]					

### Appendix B.2. Refinement Network Architecture

**Table A2:** Refinement Network Architecture.

Inputs/Branches	Operation	Kernel Size	Dilation	Stride	Output	Activation
Input	Concatenation (Input Image, Mask)					
Regular Branch	Convolution	5 × 5	1	1	32	Leaky ReLU
Regular Branch	Convolution	3 × 3	1	2	32	Leaky ReLU
Regular Branch	Convolution	3 × 3	1	1	64	Leaky ReLU
Regular Branch	Convolution	3 × 3	1	2	64	Leaky ReLU
Regular Branch	Convolution	3 × 3	1	2	128	Leaky ReLU
Regular Branch	Convolution	3 × 3	1	1	128	Leaky ReLU
Regular Branch	Atrous Convolution	3 × 3	2	1	128	Leaky ReLU
Regular Branch	Atrous Convolution	3 × 3	4	1	128	Leaky ReLU
Regular Branch	Atrous Convolution	3 × 3	8	1	128	Leaky ReLU
Regular Branch Output	Atrous Convolution	3 × 3	16	1	128	Leaky ReLU
Attention Branch Input	Concatenation (Input Image, Mask)					
Attention Branch	Convolution	5 × 5	1	1	32	Leaky ReLU
Attention Branch	Convolution	3 × 3	1	2	32	Leaky ReLU
Attention Branch	Convolution	3 × 3	1	2	64	Leaky ReLU
Attention Branch	Convolution	3 × 3	1	2	128	Leaky ReLU
Attention Branch	Convolution	3 × 3	1	2	128	Leaky ReLU
GLA Module	Attention	3 × 3	2	1	128	None
Attention Branch	Convolution	3 × 3	1	1	128	Leaky ReLU
Attention Branch Output	Convolution	3 × 3	1	1	128	Leaky ReLU
Combined Layer	Concatenation (Regular Branch Output, Attention Branch Output)					
Combined Layer	Convolution	3 × 3	1	1	128	Leaky ReLU
Combined Layer	Convolution	3 × 3	1	1	128	Leaky ReLU
Combined Layer	Upscale	Factor of 2				None
Combined Layer	Convolution	3 × 3	1	1	64	Leaky ReLU
Combined Layer	Convolution	3 × 3	1	1	64	Leaky ReLU
Combined Layer	Upscale	Factor of 2				None
Combined Layer	Convolution	3 × 3	1	1	32	Leaky ReLU
Combined Layer	Convolution	3 × 3	1	1	16	Leaky ReLU
Combined Layer	Convolution	3 × 3	1	1	3	Leaky ReLU
Refined Inpainted Image	Clamp [−1,1]					

### Appendix B.3. Discriminator Network Architecture

**Table A3:** Discriminator Architecture.

Layers	Operation	Kernel Size	Dilation	Stride	Output	Activation	Normalization
Input	Inpainted Image, Ground Truth						
Layer 1	Convolution	5 × 5	2	2	64	Leaky ReLU	Spectral
Layer 2	Convolution	5 × 5	2	2	128	Leaky ReLU	Spectral
Layer 3	Convolution	5 × 5	2	2	256	Leaky ReLU	Spectral
Layer 4	Convolution	5 × 5	2	2	256	Leaky ReLU	Spectral
Layer 5	Convolution	5 × 5	2	2	256	Leaky ReLU	Spectral
Output	Linear	1	1	1	1	None	None
